# Inducible Clindamycin Resistant Staphylococcus aureus among Patients Attending Tertiary Care Centre: A Descriptive Cross-sectional Study

**DOI:** 10.31729/jnma.6882

**Published:** 2021-11-30

**Authors:** Shanti Pradhan, Sanjib Mani Regmi, Nabina Shrestha

**Affiliations:** 1 Gandaki Medical College Teaching Hospital and Research Centre, Pokhara, Nepal

**Keywords:** *antibiotic resistance*, *clindamycin*, *methicillin-resistant Staphylococcus aureus*

## Abstract

**Introduction:**

*Staphylococcus aureus,* a superbug, resistant to multiple antibiotics led to growing interest in the usage of macrolide-lincosamide-streptogramin B antibiotics, which are now rapidly developing resistance. This study aims to find the prevalence of inducible clindamycin-resistant *Staphylococcus aureus* among obtained clinical samples from in-patient and out-patient departments of a tertiary care center.

**Methods:**

This is a descriptive cross-sectional study done in clinical samples from the in-patient and out-patient departments of a tertiary care center from September 2020-May 2021. Ethical clearance was taken from the Institutional Review Committee (Ref: 068/2077/2078). *Staphylococcus aureus* were isolated and antibiotic susceptibility tests were performed by disc diffusion method. Inducible clindamycin and methicillin resistance *Staphylococcus aureus* were detected using D-test and cefoxitin disc according to Clinical and Laboratory Standards Institute guidelines. Convenient sampling was done and the data was analyzed using Statistical Package for Social Sciences version 20. Point estimate at 95% confidence interval was calculated along with frequency and proportion for binary data.

**Results:**

Among a total of 141 *Staphylococcus aureus* isolated, the prevalence of inducible clindamycin resistant phenotype was 41 (29.1%) (21.6-36.59 at 95% Confidence Interval). Whereas, 30 (21.3%) were constitutive clindamycin resistant. The inducible 28 (47.5%) and 19 (32.2%) constitutive clindamycin resistance was higher among methicillin-resistant *Staphylococcus aureus*.

**Conclusions:**

The frequency of inducible clindamycin resistance among methicillin resistant *Staphylococcus aureus* was high, which alarms the use of macrolide-lincosamide-streptogramin B antibiotics in *Staphylococcus aureus* infections. Hence, D-test should be performed to detect inducible clindamycin resistance in routine testing to prevent treatment failure.

## INTRODUCTION

Staphylococcus aureus is an aggressive pathogen, resistant to several antibiotics that cause nosocomial as well as community-acquired infections.^1,2^ With the rise of methicillin-resistant S. aureus (MRSA), therapeutic options for S. aureus infection have narrowed.^3,4,5^

Clindamycin (CL), a macrolide, lincosamide, streptogramin B (MLS_B_) group of antibiotics is now preferred for treating methicillin-resistant S. aureus infections.^3^ However, its extensive use has led to increasing resistance among Staphylococcal strains.^6^

MLS_B_ resistance occurs frequently due to erm genes, which can be expressed inducibly (iMLS_B_, inducible Macrolide-Lincosamide-Streptogramin B phenotypes) or constitutively (cMLS_B_, constitutive Macrolide-Lincosamide-Streptogramin B phenotype).^7^ In vitro, inducible clindamycin resistant strains show clindamycin sensitive which results in treatment failure.^8,9^ Detection of iMLS_B_ can be done by D-test, which has high sensitivity and specificity.^3^ There are limited studies of the prevalence of iMLS_B_ S. aureus from Nepal.^4^

Thus, this study aims to find out the prevalence of inducible clindamycin resistant S. aureus using D-test among obtained clinical samples of a tertiary care center.

## METHODS

This is a descriptive cross-sectional study that was conducted from September 2020 to May 2021 in the Clinical Microbiology Laboratory of Gandaki Medical College Teaching Hospital and Research Centre, Pokhara, Nepal. Ethical clearance was taken from the Institutional Review Committee (Ref: 068/2077/2078). The non-repetitive clinical samples (pus, swabs, urine, blood, fluids and tips) collected aseptically; from all age and sex groups were included in the study. Specimens in leaked container, specimens of patients who did not provide consent and patients on antibiotics were excluded from the study. Convenience sampling was done and the sample size was calculated using the following formula,

n = Z^2^ × p × q / e^2^

  = 1.96^2^ × (0.152 × 0.848) / 0.06^2^

  = 138

Where,

n= sample size,
Z= 1.96 for 95% Confidence Interval (CI),
p= prevalence of study was taken from the previous study, 15.2%^3^
q= 1-p,
e= margin of error, 6%

Hence, we took 141 total isolates.

Overall 3428 samples were processed using standard microbiological techniques without delay. A total of 141 S. aureus isolates were identified by their colony characteristics, Gram staining, catalase test, slide and tube coagulase test, and growth on mannitol salt agar.^10^

Furthermore, an antibiotic susceptibility test was performed by modified Kirby Bauer's disc diffusion method on Mueller Hinton Agar (MHA) plates as per Clinical and Laboratory Standards Institute (CLSI) guidelines.^11^ S. aureus isolates with cefoxitin zone size ≥22 mm were considered methicillin-susceptible and those with ≤21mm were considered resistant. Isolates that showed erythromycin (E) resistant (zone size ≤ 13mm) and clindamycin sensitive (zone size ≥21mm) were subjected to D-test by keeping the erythromycin and clindamycin discs 15mm apart on MHA plate.^11^ Isolates with flattening of the zone of inhibition (ZOI) of clindamycin at the side adjacent to erythromycin were considered D-test positive (iMLS_B_ phenotype) whereas isolates with no flattening of ZOI around CL were D-test negative (MS phenotype). The cMLS_B_ phenotypes were resistant to both CL and E and susceptible phenotypes were sensitive to both discs. S. aureus ATCC 25923 was used to carry out quality control.

All the data collected were entered and statistical analysis was done using Microsoft Excel 2016 and Statistical Package for the Social Sciences (SPSS) version 20. Point estimate at 95% CI was calculated along with frequency and proportion for binary data.

## RESULTS

From a total of 141 clinical isolates of S. aureus tested for antibiotic susceptibility to erythromycin (E), clindamycin (CL), and other antibiotics by routine disc diffusion method, 86 (61.0%) isolates were resistant to erythromycin and 109 (77.3%) isolates were sensitive to clindamycin. The D-test performed on them showed the overall prevalence of inducible clindamycin resistance (iMLS_B_) to be 41 (29.1%) (21.6-36.59) at 95% Confidence Interval. Different types of susceptibility patterns of S. aureus isolate to clindamycin were observed. There were 30 (21.3%) isolates of constitutive resistant (cMLS_B)_ phenotype whereas there was 2 (1.4%) uncommon variant of S. aureus isolates which showed erythromycin sensitive and clindamycin resistance (E-S, CL-R) (Table 1).

**Table 1:** Distribution of MLSB phenotypes of S. aureus isolates.

Phenotypes	Total isolates n (%)
iMLSB (CL-S, E-R, and D-test positive)	41 (29.1)
cMLSB (CL-R, E-R)	30 (21.3)
MS phenotype (CL-S, E-R and D-test negative)	15 (10.6)
Sensitive (CL-S, E-S)	53 (37.6)
E-S, CL-R	2 (1.4)
Total	141 (100.0)

* S- sensitive, R- resistant

Among a total of 141 S. aureus, 59 (41.8%) isolates were MRSA while 82 (58.2%) were MSSA. Maximum number of iMLS_B_ 28 (47.5%) and cMLS_B_ 19 (32.2%), were observed among MRSA (Table 2).

**Table 2:** Distribution of S. aureus phenotypes among MRSA and MSSA.

Phenotypes	MRSA (n=59)	MSSA (n=82)	Total isolates n (%)
	n (%)	n (%)	
iMLSB	28 (47.5)	13 (15.9)	41 (29.1)
cMLSB	19 (32.2)	11 (13.4)	30 (21.3)
MS phenotype	5 (8.5)	10 (12.2)	15 (10.6)
Sensitive	6 (10.2)	47 (57.3)	53 (37.6)
E-S, CL-R	1 (1.7)	1 (1.2%)	2 (1.4)
Total	59 (100.0)	82 (100.0)	141 (100.0)

Among the S. aureus isolated from various samples, the majority of them were isolated from swab 59 (41.8%) and pus 40 (28.4%). Highest number of MRSA 28 (47.5%) and iMLS_B_ 22(53.7%) were isolated from swab samples (Table 3).

**Table 3:** Distribution of S. aureus and their different phenotypes among various clinical samples.

Samples	MRSA n (%)	MSSA n (%)	iMLSB n (%)	cMLSB n (%)	MS phenotype n (%)	Sensitive phenotype n (%)	E-S, CL-R n (%)	Total n (%)
Pus	18	22	12	4	2	22	0	40(28.4)
	(30.5)	(26.8)	(29.3)	(13.3%)	(13.3)	(41.5)	(0.0)	
Swab	28	31	22	15	7	14	1	59(41.8)
	(47.5)	(37.8)	(53.7)	(50.0)	(46.7)	(26.4)	(50.0)	
Urine	6	20	5	6	3	11	1	26(18.4)
	(10.2)	(24.4)	(12.2)	(20.0)	(20.0)	(20.8)	(50.0)	
Foley's tips	2	2	0	3	0	1	0	4
	(3.4)	(2.4)	(0.0)	(10.0)	(0.0)	(1.9)	(0.0)	(2.8)
HVS	1	1	1	0	0	1	0	2
	(1.7)	(1.2)	(2.4)	(0.0)	(0.0)	(1.9)	(0.0)	(1.4)
Blood	3	5	1	2	2	3	0	8
	(5.1)	(6.1)	(2.4)	(6.7)	(13.3)	(5.7)	(0.0)	(5.7)
Fluid	1	1	0	0	1	1	0 (0.0)	2
	(1.7)	(1.2)	(0.0)	(0.0)	(6.7)	(1.9)		(1.4)
Total	59	82	41	30	15	53	2	141
	(100.0)	(100.0)	(100.0)	(100.0)	(100.0)	(100.0%)	(100.0)	(100.0)

## DISCUSSION

S. aureus is of major concern, particularly due to its increased resistance to various antimicrobials, which has narrowed the therapeutic options for clinicians. Globally, Asia is among the region with the highest burden of MRSA.^12^ In recent days, after the burden of erythromycin resistance, clindamycin is the most desirable drug for treating S. aureus infections and thus saving the treatment option of other drugs like glycopeptides for critical cases. However, immense and irrational usage of this drug in infections has caused treatment failure in the case of inducible clindamycin phenotypes.^4,13^

Among 141 S. aureus isolates tested for inducible clindamycin resistance, 29.1% showed positive D-test (iMLS_B_ phenotype) which was similar to the study conducted by Raut et al. (25.6%) and Mohammedaman et al. (24.0%).^5,14^ Other studies reported higher incidence, 40.0% by Kumar et al. and 39.7% by Regmi et al.^2,15^ However, the lower incidence has also been reported by Adhikari et al (11.48%) and Sah et al (12%).^4,16^ As the inducible resistance was noted, it should be reported as clindamycin resistant to prevent therapeutic failure. Hence, it is necessary to perform D-test on a routine basis as false interpretation can lead to constitutive resistance among them causing therapeutic failure. There was 21.3% of cMLS_B_ S. aureus strain observed in our study which was consistent with the other studies.^2,7,17^ Therefore, the overall resistance to clindamycin in this study was73 (51.8%) which was alarmingly high. Thus, this data reflects the overuse of MLS antibiotics in our institution, which is of major concern.

The present study identified 41.8% MRSA which was in accordance with the other studies from Nepal,^3,5,18^ India^19,20^ and other countries.^7,15,21^ However, variation in the isolation rate of MRSA has been reported from different studies of Nepal^4,6,14^ and elsewhere.^2,3,15,22^ This varied frequency of the MRSA could be due to differences in the period of the surveillance-implementation of the infection control practices- and extensive use of antibiotics without prescription ahead of the definite laboratory tests. The increased frequency of MRSA could be a warning to many clinical set up like ours-where beta-lactam drugs are used injudiciously to treat bacterial infections.

The majority of the iMLS_B_ and cMLS_B_ strains were observed among MRSA (47.5% and 32.2%) than MSSA (15.9% and 13.4%) which was comparable to other studies.^5,23^ Molecular studies have stated that transposon Tn554 carried by some SCCmec elements on MRSA have ermA gene that imparts resistance to MLS antibiotics.^4^ Although few studies have reported a higher incidence of iMLSB among MSSA than MRSA.^21,24^ This study demonstrates that most the MRSA are resistant to MLS_B_ drugs-which depicts that there is low clinical efficacy of clindamycin on erythromycin-resistant MRSA. Furthermore-this study accentuates the importance of the D-test to be employed in routine antimicrobial susceptibility testing to prevent treatment failure among the cases where it is used as a substitute for other anti-MRSA drugs.

S. aureus is a skin flora that can penetrate through trivial injuries-cuts-surgical incisions- etc. In the current study- the majority of the S. aureus was isolated from swabs and pus samples. Reports from other authors have also shown similar findings.^5,25^ Thus-these evidence proves the fact that S. aureus is an important pathogen to cause pyogenic infections.

The limitation of this study was that the molecular method was not possible because of limited resources. Moreover, the molecular method could have explored the genetic characteristic of the two uncommon variants. A multicentric study within and out of this region would have strengthened the data.

## CONCLUSIONS

This study demonstrates that S. aureus is the most common pathogen causing soft tissue and wound infections. With the increase in the frequency of MRSA in our hospital setup, the treatment options for MRSA are becoming limited, as most of them are resistant to erythromycin and clindamycin in the form of iMLS_B_ and cMLS_B_. This indeed calls for the injudicious use of drugs like vancomycin and linezolid which are kept as a last resort for serious Staphylococcal infections. Therefore, there should be regular monitoring of the antimicrobial susceptibility pattern and proper enforcement of the empirical treatment protocols, to prevent antibiotic-resistant S. aureus. D-test should be mandatory on routine antibiogram testing as this will check the false clindamycin sensitive strains.
